# Woman’s Dental Association of the United States

**Published:** 1893-12

**Authors:** Eliza Yerkes

**Affiliations:** Recording Secretary


### WOMAN'S DENTAL ASSOCIATION OF THE UNITED STATES.

THE regular monthly meeting of the Woman's Dental Association of the United States was held at 1300 Arch Street, Philadelphia, October 7, 1893, Dr. Mary H. Stilwell, President, in the chair.

The minutes of the previous meeting were approved as read by the Recording Secretary.

The Corresponding Secretary announced a letter from Dr. Kate C. Moody, of Los Angeles, California, stating the cause of not sending a report to the Chicago meeting was the delay in the mail. Did not receive notice in time.

Dr. Davis, chairman of the Executive Committee, presented nine new names for membership: Drs. A. H. Graham, La Crosse, Wis.; G. S. Bright, New York City; Sarah E. Gardiner, Laramie, Wyo.; Mary Weston, Kansas City, Mo.; Ethelyn Phillips, Chicago, Ill.; Hester J. Baker, Quincy, Ill.; Cora G. Little, Holdredge, Neb.; Sarah Mary Townsend, Denver, Col.; Thelka Stein-Reuter, Madison, Wis.

On motion, reports were accepted.

A motion was made and adopted that the Recording Secretary cast the vote to elect these new applicants as members.

The President introduced Dr. C. N. Peirce, who read a paper on "Dental Diagnosis."

MRS. PRESIDENT AND MEMBERS OF THE WOMAN'S DENTAL ASSOCIATION OF THE UNITED STATES,—Before presenting the paper prepared for the evening you will allow me to congratulate you and your associates upon the position which your Association has attained, also upon its membership, and the interest which it is manifesting in professional improvement and growth. As it is sometimes profitable to recognize the previous condition and the factors which have been influential in making possible the present status, I would like to remind you that you have present this evening one whose contest with prejudice and bigotry and in favor of woman's right to a dental education made the opportunities which we are to-night enjoying not only possible but practical. Twenty-five years ago, when the first woman presented herself for matriculation in the Pennsylvania College of Dental Surgery, such a storm was created around the heads of the applicant and her friends that one might have imagined a great catastrophe was impending; but after a time the fury of the storm abated, and when the smoke had cleared away it found your guest, Professor James Truman, victorious in the right, with Henriette Hirschfeld matriculated as the first woman student in the Pennsylvania College of Dental Surgery. Since that period some sixty women have been admitted, and now the woman dental student writes her name upon the matriculation book annually, and not a ripple is heard or seen upon the quiet waters.

Such is the history of every encroachment upon time-worn customs, but it is the brave heart and clear conscience that leads the way and breasts the storm. So I congratulate you, and am happy to be with you this evening.

(For Dr. Peirce's paper, see page 889.)

DISCUSSION.

*Dr. James Truman.*—The large number of earnest women present here this evening impresses me with the fact that but a comparatively few years have elapsed since the struggle was made and finished that opened the door for women to study this profession.

The courage of the women who first essayed the *rôle* of college-students it seems to me has never received the recognition deserved. It is no slight matter to brave public opinion, and the ideas prevalent on this subject thirty years ago were altogether different from those entertained at present. Everything then stood in the way of woman's advancement in the professions and in higher education. All classes of both men and women needed to be taught that there is no sex in knowledge, and to be trained for the results of the revolution then slowly in progress.

It is, therefore, with great gratification that I am able to meet such a large body of co-workers in a calling that was once regarded, by a class of medical men, as certain to undermine the health of all the women who undertook it.

The subject presented for our consideration by Professor Peirce is one of much importance, yet I do not know that I can add anything to the very full statement he has made. It seems, however, to be true that dentistry cannot be practised if there be a neglect in the study of cases as they present. It is to be feared there is too much of routine work in practice, following day by day recognized procedures without much regard to general or systemic conditions or related facts. The origin and philosophy of pathological states need to be comprehended in every case, or failure, whole or partial, must result. Any other course is simply empiricism.

The study of irritations with their remote possible pathological results, the new foci of inflammation probable as the termination of all nerve disturbance, the action of ferments, the local and predisposing causes of disturbed function, must be sought for and properly correlated before intelligent conclusions can be reached.

The diagnosis, therefore, of the dentist cannot be limited to the oral cavity, but must comprise a general and thoughtful study of all related organs and inherited possibilities. To do this necessitates, as has been stated, education and experience; but unless the latter be combined with a study of possible contingencies and sources of disease it will not progress very far towards correct modes of treatment.

*Dr. Brubaker.*—If I have rightly comprehended the idea embodied in the writer's paper, it is that the diagnosis of any disease does not consist merely in the recognition of the pathological processes as they present themselves at the passing moment, but in the recognition also of all the antecedent causes which have been operative in the production of the pathological process, not only in the life of the individual but in the lives of his or her ancestors as well. Very frequently these causes are remote and obscure, and are to be sought for in those constitutional states or diatheses, such as the tubercular, arthritic, syphilitic, which, while active in one generation, lie dormant in the second, and reappear in some strange form in the third or fourth. This form of the general law of heredity must always be kept in mind in the investigation of the life-history of any disease. It is astonishing how frequently an apparently isolated disease has an invisible bond of connection with some ancestral disease and is dependent upon it. In order, therefore, that the diagnosis of any pathological state may be complete, and thus a satisfactory basis for a rational treatment be established, it is necessary that we possess not only a complete knowledge of its present condition but of the various phases through which it has passed, as well as those through which it will pass before it reaches its final termination. This, I conceive, would form a scientific basis for a rational system of therapeutics.

All diseases have for their production two sets of causes,—viz., internal or predisposing and external or exciting. So long as the tissues possess their normal degree of vitality or are enabled to adjust themselves to variations in external conditions, so long will health, or the physiological state, be maintained, and disease, or the pathological state, be excluded. This is, however, an ideal condition and extremely difficult of maintenance. In every disease there is a time and a cause, near or remote, for the departure from the physiological condition. I conceive it to be the writer's idea that it is the duty of the diagnostician to seek out as far as possible this time and cause to obtain a complete history of the disease. Then and then only will it become possible to explain and successfully treat many dental diseases which at present elude all the therapeutic means at our disposal.

Dr. Emily Wyeth asked Dr. Peirce about an elongated tooth that came to her care, causing serious trouble.

*Dr. Peirce.*—Mechanical force applied with the abnormal condition may result in elongation; this causes the increase of the blood to that tooth, producing the trouble.

Dr. Focht cited several cases of incorrect diagnosis, made by the non-appearance of familiar signs, and asked, "Do we devitalize enough teeth? I cannot speak from my own experience, not having been in practice long, but is capping a safe practice? As many teeth presented have been filled and capped and in years afterwards give trouble, would it not be better to devitalize at once?"

*Dr. Peirce.*—In proportion to the number of teeth capped and giving no trouble for say five or eight years and the ease enjoyed for that length of time, I think there is much in favor of capping. Of course one must take into consideration certain conditions, temperament, climate, etc.

A question arising in connection with peridental trouble, caused by absorption at the apical foramen of a superior first bicuspid, was presented by Eliza Yerkes. Treatment prescribed, crystal chloride of zinc.

Discussion and incidents of office practice by Drs. Stilwell, Davis, and Miller.

A vote of thanks was tendered Professor Peirce for his able paper.

Meeting then adjourned, to meet November 4, 1893, at the same place.

Eliza Yerkes,

*Recording Secretary.*

